# *Early*CDT®-Lung test: improved clinical utility through additional autoantibody assays

**DOI:** 10.1007/s13277-012-0379-2

**Published:** 2012-04-11

**Authors:** Caroline J. Chapman, Graham F. Healey, Andrea Murray, Peter Boyle, Chris Robertson, Laura J. Peek, Jared Allen, Alison J. Thorpe, Geoffrey Hamilton-Fairley, Celine B. Parsy-Kowalska, Isabel K. MacDonald, William Jewell, Paul Maddison, John F. R. Robertson

**Affiliations:** 1 Centre of Excellence for Autoimmunity in Cancer, The University of Nottingham, Nottingham, UK; 2 Oncimmune Ltd, Nottingham City Hospital, Nottingham, UK; 3 International Prevention Research Institute (iPRI), Lyon, France; 4 Department of Mathematics and Statistics, University of Strathclyde, Glasgow, UK; 5 Oncimmune USA LLC, De Soto, KS USA; 6 Department of Neurology, Queen’s Medical Centre, Nottingham, UK

**Keywords:** Autoantibodies, Lung cancer, Lung cancer diagnosis

## Abstract

Tumor-associated autoantibodies (AAbs) have been described in patients with lung cancer, and the *Early*CDT®-Lung test that measures such AAbs is available as an aid for the early detection of lung cancer in high-risk populations. Improvements in specificity would improve its cost-effectiveness, as well as reduce anxiety associated with false positive tests. Samples from 235 patients with newly diagnosed lung cancer and matched controls were measured for the presence of AAbs to a panel of six (p53, NY-ESO-1, CAGE, GBU4-5, Annexin I, and SOX2) or seven (p53, NY-ESO-1, CAGE, GBU4-5, SOX2, HuD, and MAGE A4) antigens. Data were assessed in relation to cancer type and stage. The sensitivity and specificity of these two panels were also compared in two prospective consecutive series of 776 and 836 individuals at an increased risk of developing lung cancer. The six-AAb panel gave a sensitivity of 39 % with a specificity of 89 %, while the seven-AAb panel gave a sensitivity of 41 % with a specificity of 91 % which, once adjusted for occult cancers in the population, resulted in a specificity of 93 %. Analysis of these AAb assays in the at-risk population confirmed that the seven-AAb panel resulted in a significant increase in the specificity of the test from 82 to 90 %, with no significant change in sensitivity. The change from a six- to a seven-AAb assay can improve the specificity of the test and would result in a PPV of 1 in 8 and an overall accuracy of 92 %.

## Introduction

Patients with lung cancer, both non-small cell lung cancer (NSCLC) and small cell lung cancer (SCLC), can mount a humoral immune response to their cancer [1–5]. Autoantibodies (AAbs) have been described not only at the time of initial diagnosis of lung cancer [1, 2], but also, in some cases, up to 5 years before the cancer is diagnosed [6–8]. There is now level 1 evidence from the US National Lung Screening Trial that earlier diagnosis saves lives; this randomized control trial reported a 20 % reduction in lung cancer mortality, following CT screening of high-risk individuals [9].

An AAb assay for lung cancer (*Early*CDT®-Lung), which was technically and clinically validated using three separate case–control study populations, has recently been reported [1, 10]. In these publications, AAbs to six tumor-associated antigens (p53, NY-ESO-1, Annexin I, CAGE, GBU4-5, and SOX2) were measured and identified up to 40 % of all lung cancers in the disease groups, with a specificity of 90 % (non-cancer controls individually matched to lung cancer sera by age, gender, and smoking history) [1, 10]. Further confirmation of the sensitivity and specificity of the test for lung cancer using four new, independent sample sets (*n* = 574 newly diagnosed lung cancers plus controls) was recently reported [11], with no significant difference in positivity for *Early*CDT-Lung among different cancer stages being seen. The performance of the test (in terms of precision and analytical linearity [10]) is such that it is now commercially available to clinicians, to assist them in the early detection of lung cancer in combination with imaging techniques.

We report here the analysis of two additional and well-described cancer-associated antigens, MAGE A4 and HuD (n-ELAV), which are known to have particular associations with lung cancer. The MAGE gene family belongs to the chromosome X-clustered cancer/testis antigens, and the members of the MAGE A family encode proteins with 50 to 80 % sequence identity to each other. The overexpression of these MAGE antigens has been described in a number of cancers including lung cancer [12, 13], and MAGE A4 has been proposed as a potential therapeutic target for immunotherapy [14]. The diagnostic potential of MAGE A4 AAb measurement has not been reported previously. HuD is a member of a family of onconeuronal RNA-binding proteins known for stabilizing RNA. It is normally expressed only on terminally differentiated neurons where it is involved in the development and maintenance of the nervous system [15–17]. Anti-Hu antibodies are often found associated with paraneoplastic encephalomyelitis or sensory neuropathy, and these antibodies have been described in neuroendocrine tumors of the lung, in particular SCLC [18–20]. In fact, 17 % of patients with SCLC have been described as having elevated levels of AAbs to HuD when compared to matched controls [20].

This manuscript reports an improved *Early*CDT-Lung panel with the addition of these two new AAb assays (i.e., MAGE A4 and HuD), and the removal of Annexin I, first in an optimization set comprised of patients with newly diagnosed lung cancer (before any treatment) and matched controls, and secondly in a prospective sample set confirming the additive value this modification brings to the original *Early*CDT-Lung panel in the clinical setting.

## Methods

### Blood samples and patient details

#### Optimization set

Serum samples from 235 patients with lung cancer (from the UK, USA, Ukraine, and Russia), obtained at or just after histopathological confirmation of the tumor, were assayed. These 235 samples represented 87 % of the lung cancers in a previously published dataset (group 3, *n* = 269) [1], which were chosen on the basis of sufficient residual sample volume being available for analysis. The lung cancers consisted of 178 NSCLCs (75.7 %), 53 SCLCs (22.6 %), and 4 others (1 sarcoma, 2× bronchogenic carcinomas, and 1 undefined lung cancer). The controls were also part of the previously published sample set and consisted of 266 healthy volunteers, 235 of which were matched to the lung cancer patients for age, gender, and smoking status. This group of controls had no evidence of any current or prior cancer including non-melanoma skin cancer. All serum samples were collected following informed consent and stored at −20 or −70°C prior to analysis. This dataset was used to re-optimize the panel performance in terms of specificity following the addition of the new antigens and the removal of Annexin I.

#### Clinical population set

The performance of the AAb test in an independent, clinically relevant sample set is reported here using the clinical samples sent for commercial *Early*CDT-Lung measurement [1, 10]. These consisted of 776 serum samples assessed by the original six-antigen AAb assay panel (May 2009–November 2010) and a further separate but consecutive 836 serum samples assessed by the updated seven-antigen AAb assay panel (November 2010–August 2011). All samples were from individuals in North America deemed by their clinician to be at an increased risk of developing lung cancer due to age and smoking history or other factors. All sera were taken under informed consent, and all patients had signed a HIPAA release, authorizing access to their medical records.

### Antigen production

Recombinant proteins were cloned into pET expression vectors (Invitrogen) and transformed into *Escherichia coli* BL21 (DE3) bacteria. The proteins p53, NY-ESO-1, CAGE, Annexin I, MAGE A4, HuD, SOX2-B, and GBU4-5 were cloned into pET21b and produced with a His tag and BirA tag [1, 10], whereas SOX2-N was cloned into pET44b and produced with a His tag and NusA tag. Negative control proteins were also produced (BirA and NusA tags alone). The recombinant proteins were expressed in BL21 (DE3) bacteria (Novagen) and grown in terrific broth (TB), autoinduction TB media (Novagen), ECPM media, or Power Broth (Molecular Dimensions). Recombinant proteins were purified by metal chelate affinity chromatography and refolded by dialysis [10, 21]. All recombinant proteins were produced by external suppliers. Quality control tests for acceptance of protein included SDS–PAGE, Western blotting with appropriate antibodies, and analytical size exclusion chromatography.

### Autoantibody detection

AAbs to the tumor-associated antigens were measured using *Early*CDT-Lung (Oncimmune USA LLC, De Soto, KS), a commercially available blood test based on ELISA principles that uses microtiter plates coated with a set of serial dilutions of recombinant antigens as previously described [10]. All assays were run blinded to the demographic data. AAbs were measured as optical density units and then expressed in calibrated reference units (RU). Positive seroreactivity for the assay was defined as (a) having evidence of a dose response to the antigen titration series and (b) an assay result above a cutoff level (described below).

### Statistics

Assay data handling (calibration of OD signal to RU) was performed by the Oncimmune LLC LIMS system. Clinical performance was expressed in terms of sensitivity (the percentage of true positives) and specificity (the percentage of true negatives). Concordance (the percentage of samples with the same test outcome in two assays being compared), accuracy (the percentage of samples correctly diagnosed), and positive predictive value (PPV; the probability of cancer given a positive test result) were also calculated. This analysis was performed using Microsoft Excel. For comparison of sensitivity and specificity values, chi-squared tests were used. Forest plots of the sensitivity at fixed specificity for subgroups were prepared using 95 % binomial confidence intervals. Similarly, for individual antigens, 95 % binomial confidence intervals were calculated for percentage positivity (sensitivity). This analysis was performed using SPSS®.

### Assessment of lung cancer risk

Underlying risk was calculated from the Spitz et al. [22] individual lung cancer risk assessment model, which captures some of the complex interactions between exposures and host susceptibility factors. The model was adapted to predict 5-year absolute risk of lung cancer, based on gender, age, and smoking history. An in-house program was used for the calculations [23].

### Optimization of assay cutoffs

A fixed target specificity of 90 % was selected for the panel of six AAb assays, and the cutoffs were obtained by optimizing sensitivity using a Monte Carlo direct search method [24] and validated as previously described [1, 10]. The method searches a random selection (*n* = 10,000) of the possible sets of cutoffs and chooses the set with the highest sensitivity for the fixed specificity. For the new panel of seven assays (including the two new antigens and SOX2-B and the removal of Annexin I), a similar Monte Carlo approach was used but this time optimizing specificity for a fixed sensitivity of approximately 40 %. The optimization was performed using R software.

### Adjustment for lung cancers in the control populations

In order to set accurate and meaningful cutoffs for lung cancer detection tests, the results obtained from a group of individuals known to have the disease must be compared with those obtained from a group of individuals with demographic and risk factors matched to the cancer group and known to be disease free. However, obtaining a truly disease-free control group is extremely problematic since CT screening studies have shown that in any high-risk group there is a small proportion of individuals harboring undiagnosed asymptomatic lung cancer [9]. The proportion of such individuals may be as high as 2.7 % in a prevalence round and 2.3 % in an incidence round (referenced in [1]). For this reason, a modified lung cancer prediction model [22] was employed that allowed for the presence of occult cancers in the control population by taking into account the most important predictors for disease such as smoking status and history as well as age. The adjustment was carried out in the Monte Carlo optimization routine as described previously [1, 24] to provide accurate sensitivity and specificity values for the *Early*CDT-Lung test.

## Results

### Optimization set

The sensitivity and specificity of the AAb assays for 235 lung cancers are shown in Table 1 where the data are also characterized by tumor type (i.e., NSCLC and SCLC), and a summary of the demographics of the population is shown in Table 2.

**Table 1:** Sensitivity and specificity of AAb assays for the optimization set

	*n*	Annexin I	p53	CAGE	NY-ESO-1	GBU4-5	MAGE A4	SOX2-B	SOX2-N	HuD	Panel of 7
All LCa	235	0 (0–2)	13 (9–18)	9 (5–13)	10 (7–15)	3 (1–7)	12 (8–17)	4 (2–8)	4 (2–8)	5 (2–8)	41 (35–48)
NSCLC	178	1 (0–3)	12 (7–17)	7 (4–11)	10 (6–16)	2 (0–5)	14 (9–20)	1 (0–3)	1 (0–3)	2 (0–5)	38 (31–46)
SCLC	53	0 (0–7)	17 (8–30)	13 (5–25)	11 (4–23)	8 (2–18)	8 (2–18)	17 (8–30)	15 (7–28)	15 (7–28)	55 (40–68)
Normals	266	0 (0–2)	3 (2–6)	1 (0–3)	2 (1–5)	2 (1–4)	4 (2–7)	1 (0–3)	1 (0–3)	1 (0–3)	9 (6–13)
Specificity	266	>99	97	99	98	98	96	99	99	99	91

Data are shown as percentage positivity following the application of the adjusted cutoffs. Numbers in parentheses are the 95 % confidence interval. Specificity for lung cancer detection in the normal population is also shown. Specificity is unadjusted for the presence of cancers in the control population

*Panel of 7* represents AAb positivity to any one of the antigens in the new seven-AAb *Early*CDT-Lung panel: p53, CAGE, NY-ESO-1, GBU4-5, MAGE A4, SOX2-B, and HuD

**Table 2:** Demographics of the optimization data set

Demographic data	Cancer sera	Normal sera
Number	235	266
Male/female	73 %/27 %	70 %/30 %
Age mean (median)	64.8 (65)	64.5 (65)
Current smoker/ex smoker	46 %/29 %	35 %/54 %
Nonsmoker/unknown	10 /15 %	11 %/0 %

Both cancer and normal sera were analyzed using both the six- and seven-AAb panel of assays. Normal sera were matched as closely as possible from the available samples to the cancer sera for age, gender, and smoking history

Elevated AAb levels to at least one of the original six antigens in the *Early*CDT-Lung test (p53, CAGE, NY-ESO-1, GBU4-5, Annexin I, and SOX2-N), using the original published cutoffs, gave an overall sensitivity for lung cancer detection of 39 % with an unadjusted specificity of 89 %, while elevated levels of AAbs to at least one of eight antigens tested (p53, CAGE, NY-ESO-1, GBU4-5, HuD, Annexin I, MAGE A4, and either SOX2-N or SOX2-B) with the new and optimized cutoffs gave an overall sensitivity for lung cancer detection of 42 % with an unadjusted specificity of 91 %. The sensitivity and specificity of the two SOX2 proteins (with a BirA or NusA tag) were assessed independently and also as part of the panel in the dataset. Concordance between the two SOX2 antigens was 99.6 % with no change in the results, suggesting either of the SOX2 proteins could be substituted in the assay.

**Fig. 1:**
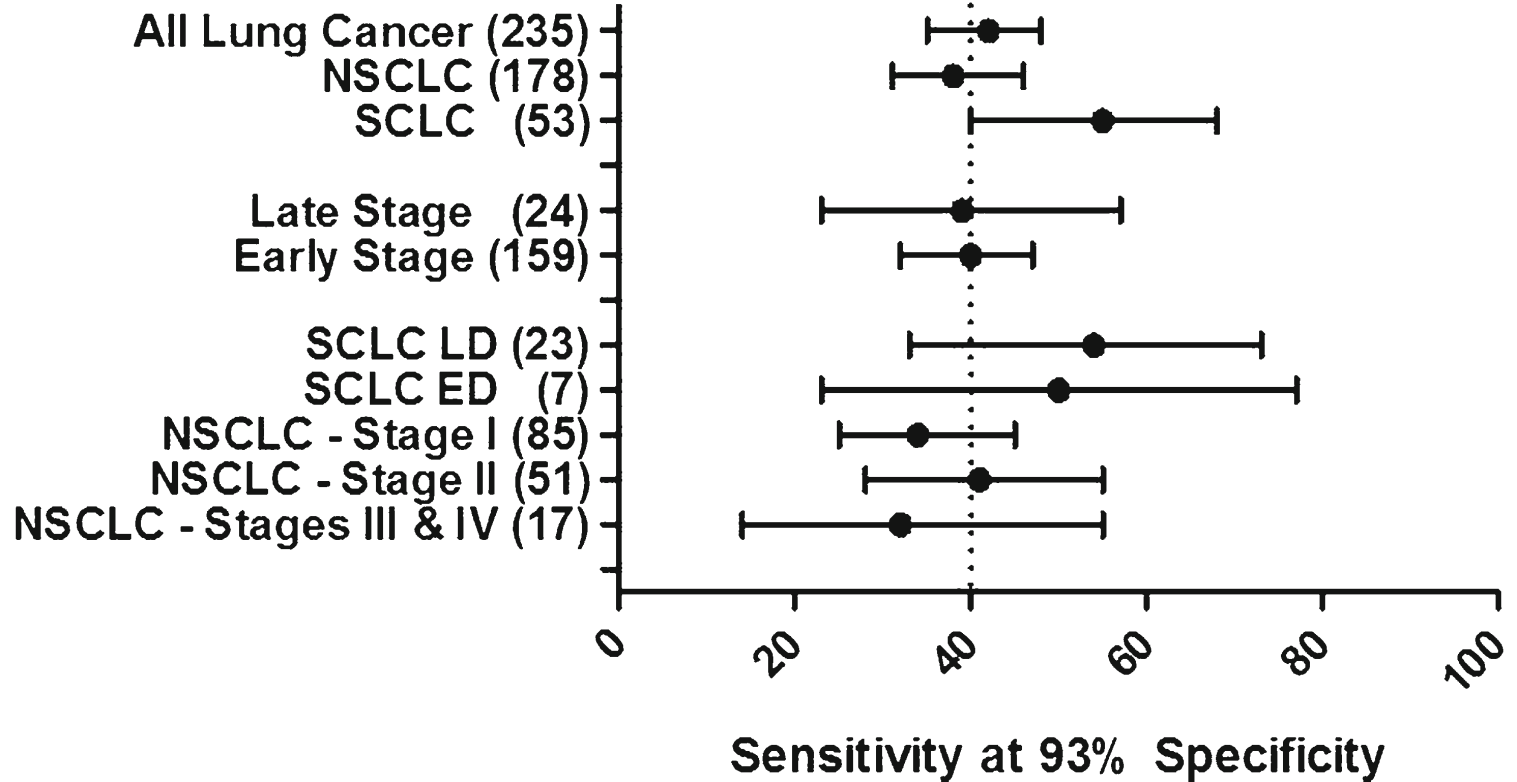
Forest plot showing the sensitivity of the *Early*CDT-Lung assay at a fixed specificity of 93 % (with confidence intervals) by tumor characteristics and lung cancer stage. Positivity is defined as having an elevated AAb assay signal to any one of the antigens in the new seven-AAb *Early*CDT-Lung panel: p53, CAGE, NY-ESO-1, GBU4-5, MAGE A4, SOX2-B, and HuD. *Vertical dashed line* represents sensitivity at 40 % (all stages of lung cancer). *NSCLC* non-small cell lung cancer, *SCLC* small cell lung cancer, *LD* limited disease, *ED* extensive disease, *early stage* stage I and II NSCLCs and LD SCLCs, *late stage* stage III and IV NSCLCs and ED SCLCs. The number of samples in each group is represented in *parentheses*

Using these optimized cutoffs, it was clear that Annexin I was no longer additive to the panel and a smaller panel of seven AAb assays (*Early*CDT-Lung (seven): p53, NY-ESO-1, CAGE, GBU4-5, MAGE A4, HuD, and SOX2-B) achieved almost identical sensitivities and specificities using these new optimized cutoffs (Table 1). This sensitivity was not dependent on the stage or grade of the cancer and was maintained at approximately 40 % even in the early-stage lung cancer samples (Fig. 1). The positivity rate for individual AAb assays in the new seven-AAb panel ranged in NSCLC from 1 (for SOX2) to 14 % (for MAGE A4) and in SCLC from 8 (for MAGE A4 and GBU4-5) to 17 % (for SOX2 and p53), with specificity for each antigen being ≥96 % (Table 1). The detection of AAbs to some antigens was, however, more specific for the detection of certain cancer subtypes; for example, MAGE A4 predicted the presence of NSCLC more often than SCLC, while the reverse was true for HuD and SOX2 (Table 1).

Allowing for the presence of potentially undiagnosed cancers in the high-risk control population (as described above), the seven-AAb test demonstrated an adjusted specificity and sensitivity of 93 and 41 %, respectively. This would mean that in a high-risk population (e.g., lung cancer prevalence of 2.4 % [25]), such a change in the panel would result in an improvement in the PPV of the test from 9 (1 in 11) to 13 % (1 in 8) and therefore an increase in the accuracy of the test from 89 to 92 %. For comparison, if a lower prevalence of lung cancer is assumed (e.g., 1.3 % [26]), the PPV of the new *Early*CDT-Lung (seven-assay test) test would be 7 % (1 in 14) with an accuracy of 92 %.

### Clinical population set

The performance of the assay was evaluated in a prospective series of individuals at increased risk of developing lung cancer, by auditing the clinical follow-up data alongside the *Early*CDT-Lung results for 1,612 clinical samples, run sequentially either on the original panel of six-AAb assays (776 samples) or the new panel of seven-AAb assays (836 samples). The two sets of commercial samples could not be analyzed by both the original and new panels so direct comparison of sensitivity and specificity could not be performed.

The demographics of the two groups were similar in terms of mean age and range; however, the proportion of men was higher in the six-AAb assay group, as was the average risk for development of a lung cancer (Table 3).

**Table 3:** Demographics of the population data sets

Demographic	Number^a^	Data
Gender		Percentage
6 AAb	776	Male 48 %, female 52 %
7 AAb	836	Male 36 %, female 64 %
Total	1,612	Male 42 %, female 58 %
Age		Mean ([(5 %ile)–median–(95 %ile)]
6 AAb	776	61 [(45)–62–(77)]
7 AAb	836	60 [(43)–59–(79)]
Total	1,612	61 [(44)–61–(78)]
Ethnicity		Percentage
6 AAb	721	Caucasian 92.0 %, Afr-Amer 5.7 %, Hispanic 1.7 %, Others 0.6 %
7 AAb	811	Caucasian 90.6 %, Afr-Amer 5.2 %, Hispanic 2.6 %, Others 1.6 %
Total	1,532	Caucasian 91.3 %, Afr-Amer 5.4 %, Hispanic 2.2 %, Others 1.1 %
Smoking		Percentage
6 AAb	770	Current 47.0 %, ex smoker 48.3 %, nonsmoker 4.7 %
7 AAb	836	Current 43.4 %, ex smoker 44.3% , nonsmoker 12.3 %
Total	1,606	Current 45.1 %, ex smoker 46.2 %, nonsmoker 8.7 %
Lung cancer risk^b^		Mean [min–(5 %ile)–median–(95 %ile)–max]
6 AAb	770	3.1 [0.0–(0.0)–2.7–(8.3)–11.9]
7 AAb	836	2.4 [0.0–(0.0)–1.6–(7.3)–11.9]
Total	1,606	2.7 [0.0–(0.0)–2.1–(8.0)–11.9]

Demographics shown for the samples run on the 6-AAb panel, 7-AAb panel, and total (where known)

*AAb* autoantibody, *Afr-Amer* African-American

^a^Number denotes numbers for which data were available

^b^Lung cancer risk was calculated according to a modified Spitz et al. lung cancer prediction model [22] based on gender, age, and smoking history

Overall, 2.7 % of these individuals (44/1,612) were diagnosed with lung cancer after having the *Early*CDT-Lung test. When the lung cancer diagnosis was analyzed according to whether the individuals were tested using the six- or seven-AAb test, 3.2 % of those who were tested using the six-AAb test and 2.3 % of those who were tested using the seven-AAb test had developed lung cancer, reflecting the increased risk calculated for the earlier group (Tables 3).

Of the 44 individuals diagnosed with lung cancer, 19 had elevated levels of AAbs, and the panel identified SCLC (1/2) and NSCLC (18/42), as well as both asymptomatic early-stage (stage IA and IB) and later-stage disease. Using the original panel of six-AAb assays and original cutoffs generated in our previous publication [10], the specificity/sensitivity in the first set of 776 samples was 82 %/40 %. Using the new panel of seven-AAb assays and the cutoffs established in the optimization set, the specificity/sensitivity in the second set of 836 samples was 90 %/47 % (Table 4). This change from the original six-AAb to the new seven-AAb panel represented a significant improvement in the specificity of the test for cancer detection (*p* < 0.0001) with no significant difference between the sensitivity of the two panels (*p* = 0.63; probably due to small numbers). Assuming the calculated risk of developing lung cancer for each group was 3.1 and 2.4 %, respectively (Table 3), this would confer an increase in the PPV of the test from 1 in 15 to 1 in 10.

**Table 4:** Audit of *Early*CDT-Lung test

	Number of participants	Confirmed lung cancers^a^, *N* (%)	No lung cancer diagnosis^b^, *N* (%)
Panel of 6-AAb assays			
Total	776	25 (3.2)	751 (96.8)
Positive AAb assay result	145	10 (6.9)	135 (93.1)
Negative AAb assay result	631	15 (2.4)	616 (97.6)
Overall panel sensitivity or specificity		Sensitivity 40 %	Specificity 82 %
Panel of 7-AAb assays			
Total	836	19 (2.3)	817 (97.7)
Positive AAb assay result	87	9 (10.3)	78 (89.7)
Negative AAb assay result	749	10 (1.3)	739 (98.7)
Overall panel sensitivity or specificity		Sensitivity 47 %	Specificity 90 %

Original six-AAb assay panel (performed on 776 samples) and new seven-AAb assay panel (performed on 836 samples) showing the number of samples that were identified as being positive or negative in the *Early*CDT-Lung test and the number of confirmed cases of lung cancer. Panel of 6 represents AAb positivity to any one of the original six-AAb *Early*CDT-Lung panel: p53, CAGE, NY-ESO-1, GBU4-5, Annexin I, and SOX2-N. Panel of 7 represents AAb positivity to any one of the new seven-AAb *Early*CDT-Lung panel: p53, CAGE, NY-ESO-1, GBU4-5, MAGE A4, SOX2-B, and HuD

^a^Number of lung cancers detected—correct as of August 2011 following CT and biopsy

^b^Number of individuals assessed as being free from lung cancer, as of August 2011

## Discussion

Previous publications using validated, calibrated assays have confirmed the utility of measuring AAbs to tumor-associated antigens as an aid for the identification of early-stage lung cancers [1, 11]. The data presented in this manuscript reveal that improvements of such a test can be achieved by adding two new antigens and dropping one (now redundant) antigen from the *Early*CDT-Lung panel, and re-optimizing the cutoffs. This change essentially maintained the previously reported 40 % sensitivity of the test for lung cancer [1] even for early-stage more treatable disease. Importantly however, it improved the specificity of the test (once adjusted for occult cancers in the population) from 90 % as previously reported [1] to 93 % in the same retrospective case–control (optimization) set. In a clinical setting, such an improvement would result in an increase in the PPV of the test and a 30 % reduction in “false” positive tests, important benefits to both patients and clinicians.

Since the two additional antigens were added to ultimately increase the specificity of *Early*CDT-Lung test, it was deemed appropriate to report the performance of the test in a clinical setting, where individuals at an increased risk of developing lung cancer were tested. Data from an audit of the first 1,612 samples run on the *Early*CDT-Lung test revealed that the performance of the test was as expected in a clinically relevant group of individuals at an increased risk of developing lung cancer, and the clinical results mirrored differences in the actual (as of August 2011) and calculated (Spitz model [22]) risk between the two groups. A difference in the gender proportion between the two clinical groups was noted; however, there are no reports of differences in autoantibody levels in individuals with lung cancer between genders [1]. Furthermore, a recent study of the demographics of normal individuals also showed no difference in autoantibody levels due to gender or ethnicity in a normal group [27].

Analysis of the performance of the *Early*CDT-Lung test in the clinical population dataset showed that the sensitivity of the test for lung cancer, reported in the optimization set, was maintained in the clinical setting, where at least 40 % of the lung cancers had a positive test. Although the number of lung cancers in the audit was relatively small, both panels were successful in detecting early-stage disease.

The greatest impact seen with the new seven-AAb panel was the highly significant improvement in the specificity of the test in the clinical setting. While in the retrospective case–control set the improvement in the assay specificity resulted in a 30 % reduction in false positives, in the prospective clinical audit data, the change to the seven-AAb panel resulted in a 44 % reduction in the number of “false positive” tests. This is because in the clinical population, the specificity of the six-AAb panel was lower than expected at 82 %, while the seven-AAb panel revealed a specificity of 90 % (unadjusted for occult cancers), a level similar to that predicted from the optimization dataset. An individual predicted to be at an increased risk of lung cancer due to demographic risk factors including smoking history, gender, and age, and who then had a positive *EarlyCDT*-Lung test, would be at a higher risk for harboring lung cancer than predicted; with the introduction of the seven-AAb version of the test, this increase in risk is even greater.

The seven-AAb test with a specificity/sensitivity of 93 %/41 % in a high-risk population (e.g., prevalence of 2.4 % [25]) has an overall accuracy of 92 % compared to approximately 50 % for CT [28]. The authors, however, view AAb technology and CT imaging as being complementary rather than competitive and that the presence of AAbs may provide an aid to early detection of lung cancer, particularly in early-stage disease which is potentially curable. This improved test may therefore prove useful in the management of high-risk individuals.
